# Adipose tissue aging partially accounts for fat alterations in HIV lipodystrophy

**DOI:** 10.1080/21623945.2022.2042962

**Published:** 2022-03-17

**Authors:** Pere Domingo, Marta Giralt, Aleix Gavaldà-Navarro, Albert Blasco-Roset, Alejandro Delgado-Anglés, José Miguel Gallego-Escuredo, Maria Del Mar Gutiérrez, Maria Gracia Mateo, Rubén Cereijo, Joan Carles Domingo, Francesc Villarroya, Joan Villarroya

**Affiliations:** a Infectious Diseases Unit and Institut de Recerca Hospital de la Santa Creu I Sant Pau, Barcelona, Spain; b Departament de Bioquímica I Biomedicina Molecular and Institut de Biomedicina (IBUB), Universitat de Barcelona, Barcelona, Spain; c Ciber Fisiopatología de la Obesidad Y Nutrición, Barcelona, Spain; d Institut de Recerca Sant Joan de Déu Barcelona, Spain

**Keywords:** Lipodystrophy, adipose, HIV, ageing, autophagy, inflammation

## Abstract

Lipodystrophy is a major disturbance in people living with HIV-1 (PLWH). Several systemic alterations in PLWH are reminiscent of those that occur in ageing. It is unknown whether the lipodystrophy in PLWH is the consequence of accelerated ageing in adipose tissue. We compared systemic and adipose tissue disturbances in PLWH with those in healthy elderly individuals (~80 y old). We observed similarly enhanced expression of inflammation-related genes and decreased autophagy in adipose tissues from elderly individuals and PLWH. Indications of repressed adipogenesis and mitochondrial dysfunction were found specifically in PLWH, whereas reduced telomere length and signs of senesce were specific to elderly individuals. We conclude that ageing of adipose tissue accounts only partially for the alterations in adipose tissues of PLWH.

## Introduction

Lipodystrophy has been a major disturbance occurring in people living with HIV-1 (PLWH) under antiretroviral treatment. Lipodystrophy in PLWH is characterized by a complex set of alterations in adipose tissue that involve peripheral lipoatrophy (i.e., loss of subcutaneous adipose tissue in the face, arms, and legs) often associated with visceral fact lipohypertrophy [1]. This is associated with enhanced cardiovascular risk and altered systemic metabolism. The recent use of less-toxic antiretroviral drugs (basically withdrawal of thymidine analogues from the antiretroviral drug patterns of treatment) has decreased the frequency of overt peripheral lipoatrophy, although signs of fat alterations remain in PLWH [2]. Patients who received thymidine analogues may have persistent fat alterations several years after therapy discontinuation [3], and young adults who were infected with HIV during childhood and exposed to older-generation antiretroviral drugs appear to be particularly susceptible to persistent lipoatrophy after discontinuation [4].

Multiple non-infectious comorbidities involving metabolic, neurological, and cardiovascular systems occurring in PLWH have been said to be reminiscent of those that appear progressively with ageing in the non-infected population [5]. The concept of ‘accelerated ageing’ has been proposed to describe the set of comorbidities appearing in PLWH [6]. Ageing profoundly alters adipose tissue [7,8] and there is a redistribution of fat depots in the elderly, with subcutaneous depots loss at the expense of increased visceral fat [9]. Advanced ageing is associated with a loss of facial adipose tissue that is reminiscent of the facial lipoatrophy seen in PLWH [10]. Moreover, adipose tissue may be a site of the so-called ‘inflammaging’ phenomenon: a mildly increased pro-inflammatory status intrinsically associated with ageing [11]. Decreased adipogenic capacity, reduced autophagy, and increased cellular senescence have been proposed to take place in ageing adipose tissue [12,13]. It has been proposed that HIV-associated lipodystrophy may be related to accelerated ageing elicited by antiretroviral drugs and HIV infection-associated events [14]. To ascertain whether accelerated adipose tissue ageing may be a key pathogenic component in HIV lipodystrophy, we herein compared alterations in biomarkers of inflammation, adipogenesis, senescence, and autophagy in sera and adipose tissue samples obtained from aged individuals with no overt pathology and from PLWH with lipodystrophy.

## Materials and methods

Three groups were enrolled in this cross-sectional study: (a) healthy individuals over 80 y old (n = 28); (b) HIV-infected, antiretroviral-treated patients with lipodystrophy (PLWH-LD) (n = 60), (mean age, 46 y) (see Supplementary Table 1 for treatment); and (c) 34 healthy controls age-matched with the PLWH-LD group. An elderly patient was considered healthy when there was no evidence of any chronic disease, including inflammatory disease, cardiovascular disease, hepatitis, liver insufficiency, fever of undetermined origin, diabetes mellitus, and cancer. Only arterial hypertension and antihypertensive therapy were allowed. Lipodystrophy in patients was assessed according to Lichtenstein et al. [15]. Briefly, the degree of lipodystrophy in PLWH was rated at distinct anatomical sites (face, arms, buttocks, legs, abdomen, neck, and breasts) as absent (score of 0), mild (noticeable on close inspection; score of 1), moderate (readily noticeable by patient and/or physician; score of 2), or severe (readily noticeable to a casual observer; score of 3). A clinical diagnosis of lipodystrophy was given to a patient with an overall score 17. In addition, patients with severe fat changes in one body location were considered to be lipodystrophic. The overall exclusion criteria included body mass index (BMI) >30 kg/m^2^, anticoagulant treatment, oral antidiabetic therapy, and hormonal treatment. This study was approved by the ethics committee of Hospital de la Santa Creu i Sant Pau, Barcelona, and all patients provided informed written consent.

BMI was calculated, and waist circumference was measured to the nearest millimetre using anatomical landmarks, as defined by the Third National Health and Nutrition Evaluation Survey. Whole-body, dual-energy X-ray absorptiometry scans (HologicQDR-4500A; Hologic, Inc., Waltham, MA, USA) were conducted by a single operator and used to determine the body fat content. Plasma and serum were obtained from blood drawn from seated patients after a 12-hour overnight fast and at least 15 minutes after the placement of a peripheral intravenous catheter. All lipid measurements were performed using a Hitachi 911 system (Roche Diagnostic Systems, Basel, Switzerland). Insulin resistance was estimated by the homoeostasis model assessment method (HOMA-IR). Laboratory procedures for determination of clinical biochemistry have been described elsewhere [16]. Circulating TNFα, IL6, IL8, HGF, NGF, MCP1, FGF21 and leptin levels in serum were measured using an antibody-linked, fluorescently labelled microsphere bead-based multiplex analysis system (Linco Research/Millipore, Billerica, MA, USA) and quantified using Luminex100ISv2 equipment. Adiponectin levels were determined by ELISA (Merck Life Science, Madrid, Spain).

Biopsy samples of subcutaneous fat from adults were obtained through a small surgical biopsy performed by an 8-mm punch under local anaesthesia with mepivacaine. Samples from newborns (n = 5) with a gestational age of 25–39 weeks who survived for no more than 24 hours post-partum have been described in detail elsewhere [17]. Tissue samples were immediately frozen and stored at −80°C. Each sample was homogenized in RA1 buffer, and RNA was isolated using a column-affinity-based methodology that included on-column DNA digestion (Macherey-Nagel, Düren, Germany). One microgram of RNA was transcribed to cDNA using MultiScribe reverse transcriptase and random-hexamer primers (TaqMan Reverse Transcription Reagents; Applied Biosystems, Foster City, CA, USA). For quantitative mRNA expression analysis, TaqMan reverse transcriptase (RT)-polymerase chain reaction (PCR) was performed on an ABI PRISM 7700HT sequence detection system (Applied Biosystems). The TaqMan RT-PCR reactions were performed in a final volume of 25 µl using TaqMan Universal PCR Master Mix, No AmpErase UNG reagent, and the primer pair probes listed in Supplementary Table 2. Controls with no RNA, primers, or RT were included in each set of experiments. Each sample was run in duplicate, and the mean value of the duplicate results was used to calculate the mRNA levels for the genes of interest. Expression levels of gene transcripts were considered negligible when, under the above standard RT-PCR conditions, the cycle threshold was >40. Values were normalized to that of the reference control (18S ribosomal RNA) using the comparative 2^−ΔCT^ method, following the manufacturer’s instructions. Parallel calculations performed using the reference gene, PPIA, yielded essentially the same results.

For quantification of specific protein levels, adipose tissue samples were homogenized in cold buffer (10 mM HEPES, pH 7.5, 5 mM EDTA, 5 mM dithiothreitol, 5 mM MgCl2), and a protease inhibitor cocktail (Complete-mini; Roche, Sant Cugat, Spain). For Western blot analysis, homogenates containing 40 µg of protein were mixed with equal volumes of 2× sodium dodecyl sulphate (SDS) loading buffer, incubated at 90°C for 5 min, and electrophoresed on SDS/polyacrylamide gels. After undergoing transfer to Immobilon-P membranes (Millipore, Billerica, MA, USA), the proteins were probed using a primary antibody against LC3B (Cell Signalling, Leiden, The Netherlands) and monoclonal anti-p53 (Thermo Fisher Scientific), detected using a goat anti-rabbit or anti-mouse HRP-conjugated secondary antibodies (Santa Cruz Biotechnology, Dallas, TX, USA), and visualized using ECL reagents (Immobilon Western; Millipore, Germany). Membranes were stained with Coomassie blue (Sigma-Aldrich, St. Louis, MO, USA) to normalize the amount of protein loaded. The Multi-Gauge software (Fujifilm) was used for densitometric analyses (3–6 individual samples were analysed per group).

For telomere length measurements, DNA was isolated from the tissue homogenate used for RNA extraction, dissolved in 70% ethanol, and purified by phenol/chloroform extraction. Telomere length was assessed by real-time PCR essentially as described previously [16,18]. Briefly, PCR was performed using 35 ng of total DNA (PCR template) in a reaction containing 10 μL of 2x SYBR Green Master Mix reagent (Applied Biosystems) and 100 nM of specific primers (Sigma) for telomere repeats (Tel) or the single-copy housekeeping gene, 36B4 (acidic ribosomal phosphoprotein P0), in a final volume of 20 μl (See Supplementary Table 3 for primer sequences). CT values were obtained for both Tel and 36B4 in each sample. Relative telomere abundance was quantified as the Tel/36B4 ratio using the comparative 2^−ΔCT^ method.

For statistical analysis, significance was assessed using one‐way ANOVA followed by Tukey’s post-hoc test, or the two‐tailed unpaired Student’s *t*‐test. Data are expressed as means ± standard error of the mean (s.e.m.), and statistical significance was set when P < 0.05. The GraphPad Prism 6 statistical software (GraphPad, San Diego, CA, USA) was used. Discrepancies among standard deviations of experimental groups were assessed with the Brown–Forsythe test or *F*‐test as appropriate. Welch’s correction was applied whenever unequal variances were detected. Outliers were detected and removed prior to significance analyses using Grubbs’ test.

## Results

Anthropometric, body composition, and metabolic parameters from PLWH patients under antiretroviral treatment showing lipodystrophy, including peripheral lipoatrophy (LD group), elderly individuals (mean age, 80 y) with no overt pathology; and control healthy individuals of a similar age range as those in the PLWH group (C group) are shown in Table 1. BMI was similar in the LD and C groups and significantly increased in the elderly group. The trunk/appendicular fat ratio, indicating asymmetry of fat distribution, was highly increased in the LD patients. The elderly group showed trunk/appendicular fat ratios and waist-to-hip ratios that were intermediate between the C and LD groups.

**Table 1.** Demographic, metabolic, anthropometric and body composition markers

	**Controls** (*n* = 34)	**Elderly** (*n* = 28)	**PLWH-LD** (*n* = 60)	*P* value
Sex (no. of men (%))	25 (73.5)	14 (50)	43 (71.7)	
Age	40.82 ± 0.52	80.78 ± 1.14***	46.27 ± 1.05***###	<0.0001
Weight	74.34 ± 2.12	73.32 + 2.68	66.77 ± 1.65*	0.01
BMI	24.54 + 0.46	28.35 + 0.76***	24.10 ± 0.45###	<0.0001
Waist to Hip ratio	0.87 ± 0.01	0.92 ± 0.01**	0.95 ± 0.01***	<0.0001
Glucose (mmol/l)	4.84 ± 0.07	5.85 ± 0.21***	5.66 ± 0.15***	0.0001
Insulin (pmol/l)	40.28 ± 4.39	211.30 ± 20.51***	100.10 ± 8.68***###	<0.0001
HOMA-r	0.77 ± 0.08	2.61 ± 0.20*	1.98 ± 0.18***	<0.0001
Bilirubin (mmol/l)	8.00 ± 1.32	11.86 ± 0.94	8.46 ± 1.32###	0.04
AST	20.56 ± 1.17	21.47 ± 1.24	31.35 ± 1.9***###	<0.0001
ALT	23.76 ± 2.36	18.16 ± 1.00	37.32 ± 3.15**###	<0.0001
Triglycerides (mmol/l)	0.89 ± 0.06	1.03 ± 0.07	2.25 ± 0.17***###	<0.0001
Cholesterol (mmol/l)	5.16 ± 0.16	5.58 ± 0.17	5.20 ± 0.19	0.30
HDL (mmol/l)	1.48 ± 0.05	1.84 ± 0.06***	1.23 ± 0.04**###	<0.0001
LDL (mmol/l)	3.28 ± 0.15	3.73 ± 0.17	2.94 ± 0.15##	0.002
	(n = 9)	(n = 14)	(n = 20)	
Total body fat (%)	26.31 ± 2.12	37.87 ± 1.41***	20.96 ± 1.57###	<0.0001
Trunk fat (kg)	9.59 ± 0.89	15.67 ± 1.05**	8.56 ± 1.05###	<0.0001
Appendicular fat (kg) Trunk/appendicular fat ratio	6.70 ± 0.62 1.48 ± 0.15	8.25 ± 0.35 2.03 ± 0.69	2.51 ± 0.32***### 4.11 ± 0.56**##	<0.0001 0.0005

Parameters are expressed as mean ± SEM unless specified. *P* values were calculated using one-way analysis of variance and Tukey post-test for parametric data. Comparison with Healthy is *P* < 0.05; ** when *P* < 0.01; *** when *P*< 0.001; # is shown when comparison with Elderly is *P* < 0.05; ## when *P* < 0.01; and ### when P < 0.001. BMI, body mass index; HDL, high-density lipoprotein, LDL, low-density lipoprotein, HOMA-r, homoeostasis model assessment of insulin resistance.

Glycaemia, insulin, and the HOMA-IR index were similarly increased in elderly and LD patients relative to controls (Table 1), indicating that although there was no overt diabetes in these groups, they showed similar alterations in glucose/insulin homoeostasis that are reminiscent of insulin resistance. Biomarkers of hepatic damage (ALT, AST) and triglyceridemia were increased significantly only in the LD group.

**Figure 1.**
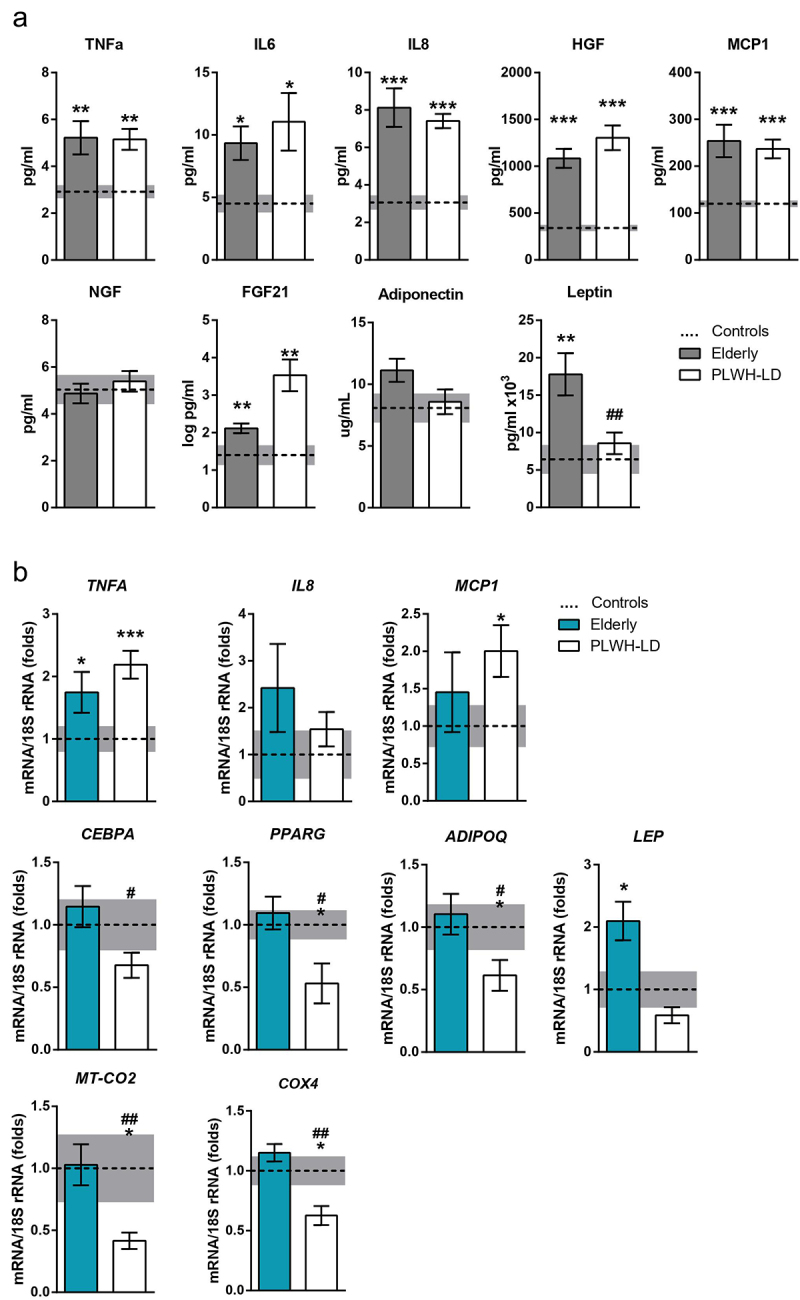
**Circulating and transcript levels of inflammatory and adipose cytokines in elderly healthy and PLWH individuals with lipodystrophy, compared to healthy young controls**. (a) Circulating levels of pro-inflammatory and metabolism-related cytokines in plasma from controls (*n* = 34), elderly (*n* = 28), and people living with HIV with lipodystrophy (PLWH-LD) (*n* = 60). (b) Proinflammatory and adipose metabolism-related mRNA levels in adipose tissue samples from the same three groups. The bars represent means ± SEM. Dotted line and grey bar represent mean ± SEM in healthy controls. (*p < 0.05, **p < 0.01, ***p < 0.001 compared with controls; #*p* < 0.05, ##*p* < 0.01 comparing PLWH-LD with elderly).

The levels of the pro-inflammatory cytokines, tumour necrosis factor alpha (TNFα), interleukin-(IL)-6, IL-8, and monocyte chemoattractant protein-1 (MCP-1), as well as hepatocyte growth factor (HGF), were increased to similar extents in the LD and elderly groups relative to the young healthy controls (Figure 1a). Leptin levels were specifically increased in the healthy elderly patients, and those of fibroblast growth factor 21 (FGF21) were significantly increased in both LD and elderly individuals. The adiponectin/leptin ratio, a proposed index of adipose tissue dysfunction [19,20], was significantly lower in elderly individuals (0.69 ± 0.12, *P = 0.039) than in young controls (1.26 ± 0.30) and LD patients (1.11 ± 0.42).

Figure 1b shows the expression levels of gene sets related to inflammation, adipose function, and mitochondrial function in subcutaneous adipose tissue. For inflammation, the mRNA levels of TNFα (*TNFA*) were increased in both elderly and LD individuals. The mRNA level of MCP1 (*MCP1*) showed an increasing trend in the elderly and was significantly up-regulated in LD patients. The expression levels of the genes encoding the master adipogenesis transcription factors, CCAAT-enhancer-binding protein alpha (*CEBPA*) and peroxisome proliferator-activated receptor gamma (*PPARG*), were specifically reduced in the LD group. A similar expression profile was found for the gene encoding adiponectin. Leptin gene expression was significantly increased only in adipose tissue from the elderly group. For marker genes of mitochondrial function, the transcript levels of both cytochrome oxidase subunit-2 (*MT-CO2*, mitochondrial genome-encoded) and subunit-4 (*COX4*, nuclear-encoded) were significantly reduced in the LD group.

**Figure 2.**
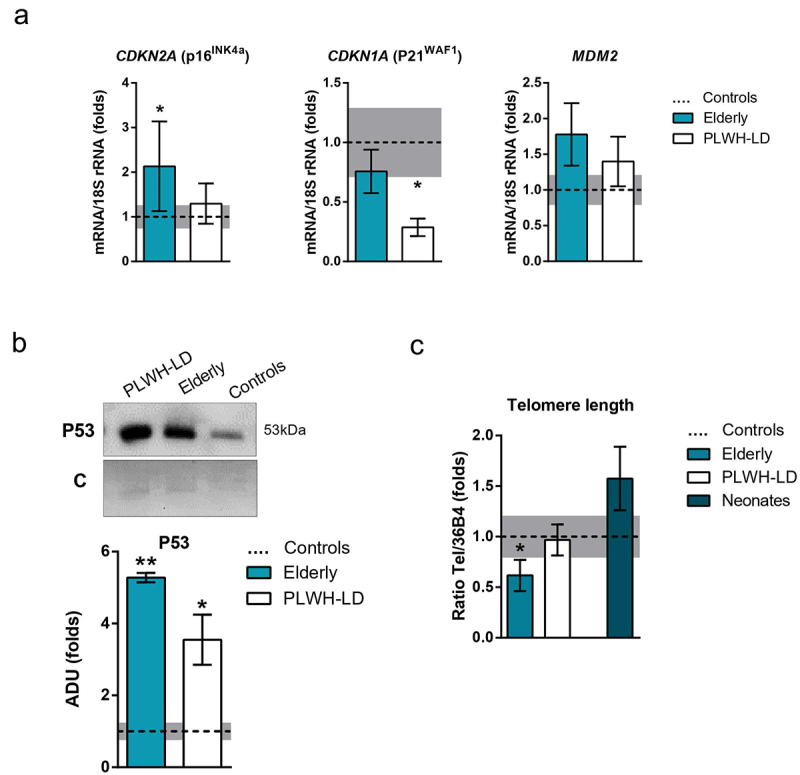
**Senescence markers in elderly healthy and PLWH individuals with lipodystrophy, compared to healthy young controls**. (a) mRNA levels of *CDKN1A, CDKN2A*, and *MDM2* in adipose tissue samples from controls (*n* = 10), elderly (*n* = 12), and PLWH-LD (*n* = 18). (b) Representative image and quantification of P53 protein levels in control, elderly, and PLWH-LD adipose tissue samples (*n* = 3–4). C: Coomassie blue staining. (c) Telomere length measurements in adipose tissue samples from the same three groups, also including a subset of neonate samples (*n* = 5). The bars represent means ± SEM. Dotted line and grey bar represent mean ± SEM in healthy controls. (*p < 0.05, **p < 0.01 compared with controls).

Next, we analysed several parameters that are directly indicative of ageing processes in adipose tissue. Among biomarkers for senescence, the expression of *CDKN2A* (encoding p16^INK4a^) was significantly increased only in adipose tissue from elderly individuals (Figure 2a). In contrast, the expression of *CDKN1A* (encoding p21^WAF−1^) was significantly decreased only in the LD group. P53 protein levels were similarly increased in adipose tissue from elderly and LD individuals relative to young healthy controls (Figure 2b) whereas the gene expression levels of murine double minute-2 (MDM2), a negative regulator of p53, were not statistically different in adipose tissue from elderly or LD individuals relative to controls.

Telomere length tended to be decreased in adipose tissue from elderly patients relative to young healthy controls, but not to a statistically significant degree. We assessed a set of fat samples from neonates, with the goal of validating our observations. We found that the telomeres length was significantly shorter in adipose tissue from the elderly group relative to neonates, while that in LD patients was similar to the age-matched healthy control group (Figure 2c).

**Figure 3.**
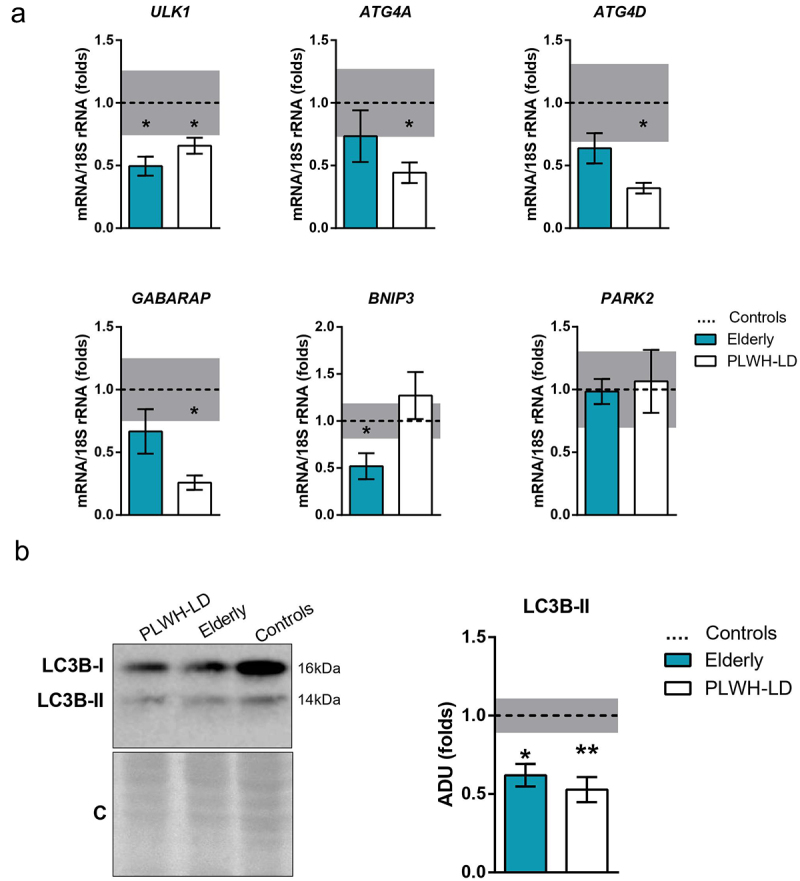
**Autophagy markers in elderly healthy and PLWH individuals with lipodystrophy, compared to healthy young controls**. (a) Gene expression levels of autophagy markers in adipose tissue samples from controls (*n* = 10), elderly (n = 12), and PLWH-LD (*n* = 18). (b) Representative image and quantification of LC3B-II protein levels in control, elderly, and PLWH-LD adipose tissue samples (*n* = 3–6). C: Coomassie blue staining. The bars represent means ± SEM. Dotted line and grey bar represent mean ± SEM in healthy controls. (*p < 0.05, **p < 0.01 compared with controls).

We further analysed several parameters related to autophagy, a process known to be altered with the ageing of tissues, including fat [21]. The levels of transcripts encoding proteins involved in autophagy (*ULK1, ATG4A, ATG4D, GARABP*) trended lower in the elderly and LD groups relative to healthy controls (Figure 3a); this down-regulation was systematically significant in the LD group, but significant only for *ULK1* in the elderly group. The transcript level of *BNIP3* was specifically down-regulated in elderly individuals. The expression of *PARK2*, encoding the mitophagy-related protein parkin, was unaltered. The protein levels of LC3B-II, which is used to indicate the autophagy status of a tissue, were down-regulated in elderly and LD patients relative to healthy controls (Figure 3b).

## Discussion

Healthy elderly individuals show alteration in the symmetry of adipose distribution, with the subcutaneous depot and central fat being somewhat decreased and increased, respectively; these changes were milder than those seen in PLWH, but were reminiscent of lipodystrophy, as previously reported in advanced ageing [9]. We observed remarkably similar alterations in the circulating parameters of cohorts representing PLWH with lipodystrophy and elderly individuals, including mild increases in insulin resistance and increased levels of proinflammatory cytokines and FGF21. These alterations in systemic parameters had been previously shown for both groups in independent reports [22–27]. Our direct comparative study confirms the previous findings and shows that there is remarkable similarity in the extents of these disturbances between elderly individuals and PLWH with lipodystrophy.

We found mild but concordant increases in pro-inflammatory gene expression levels in subcutaneous fat from PLWH and elderly individuals. However, the indications of impaired adipogenesis (decreased *CEBPA* and *PPARG* expression) and altered mitochondrial function (decreased cytochrome oxidase subunit gene expression) found in patients with lipodystrophy, which were consistent with previous reports [24], were not present in adipose tissue from the elderly. The induction of a mild systemic and local pro-inflammatory environment has been proposed to account for repressed adipogenesis in subcutaneous fat from PLWH with lipodystrophy [24,28]. The finding that pro-inflammatory cytokines were altered similarly between PLWH and elderly individuals but adipogenesis was altered only in PLWH seems to suggest that insults other than inflammation may be related to lipoatrophy in PLWH. On the other hand, the known mitochondrial toxicity elicited by antiretroviral drugs, which promotes lipodystrophy [29], may account for the PLWH-specific alteration in the expression of genes encoding mitochondrial proteins. An exception is leptin, whose adipose tissue gene expression and circulating levels were increased specifically in the elderly. This was reported previously in aged individuals [30] and is likely to be due to the increased percentage of total adipose tissue body content that is found in the elderly, often at the expense of reduced muscle mass (reference 9 and current results). This phenomenon is not seen in PLWH with lipodystrophy. Because of leptin increase, the adiponectin/leptin ratio, a recently proposed indicator of adipose tissue dysfunction [19,20], is specifically decreased in the elderly individuals. This could indicate the existence of ageing-related dysfunction in ageing, potentially related to insulin resistance and inflammation, not shared by PLWH. However, this ratio has not been yet validated in relation to ageing and such interpretation should be taken with caution.

Our analysis of parameters strictly related to biological ageing revealed some alterations specifically in elderly individuals, whose adipose tissue showed increased gene expression of *CDKN2A* (encoding p16^INK4a^, a marker of senescence) and reduced telomere length, consistently with previous reports [12,31]. Adipose tissue from PLWH did not show such alterations. In contrast, p53, which is up-regulated in mouse models of adipose tissue ageing [32], is similarly increased in adipose tissues from elderly and PLWH individuals and, according to our results, this is not attributable to major alterations in the expression of its negative regulator MDM2. This indicates that adipose tissue from PLWH shows some signs of intrinsic biological ageing, but not extensive. Increased levels of p16 was reported in adipose tissue from four HIV patients with lipodystrophy treated with protease inhibitor-type antiretroviral drugs [33]. Our cohort of PLWH in whom adipose tissue was analysed (18 individuals) included only 10 patients whose antiretroviral treatment had included protease inhibitors. This may explain the discrepancy between our results and that previous report. Treatment with protease inhibitors may account for the repression of expression of marker genes of adipogenesis in PLWH, given the potential effects of these antiretroviral drugs impairing adipocyte differentiation [34–36]. However, other drug components such as reverse transcriptase inhibitors and even HIV-encoded proteins may also be involved in such repression of adipogenesis [37]. The expression level of *CDKN1A* (encoding p21^WAF−1^, a biomarker of cellular senescence *in vitro*) was unaltered in adipose tissue from elderly individuals, in accordance with ageing-related findings in other tissues [38].

We found a concordant reduction of autophagy-related parameters in adipose tissue from PLWH and elderly individuals. Autophagic activity is known to decrease with ageing in multiple tissues [39], possibly including adipose tissue [13], and ageing-associated diseases are often ascribed to this decline in autophagy. Our observations suggest that the adipose tissue dysfunction associated with the ageing-related decline in autophagy may also be taking place in fat from PLWH developing lipodystrophy. Noteworthy, thymidine analogues are among the antiretroviral drugs that are more prone to induce lipodystrophy, and have been reported to repress autophagy in adipocytes *in vitro* [40].

There are some limitations to our study. The relatively moderate number of elderly patients, due to the obvious difficulties in recruiting very old healthy individuals volunteering for fat biopsies, ethics constrains as well as the small size of biopsies in this group precluded the study of a more extensive panel of parameters. Moreover, this limitation also precluded performing dynamic studies related to autophagy flux or mitochondrial respiratory activity that would be more informative than current data based on steady-state levels of transcripts and proteins. However, the value of the ultimately significant number of individuals enrolled in the healthy elderly group, the determination of DEXA-based adipose tissue parameters, and access to adipose tissue samples for comparative purposes with LD patients are all strengths of the study.

## Conclusions

Our study identifies several processes that are concomitantly altered in adipose tissue from healthy elderly individuals and PLWH patients with lipodystrophy (inflammation, decreased autophagy) as well as processes that are not concomitantly altered (repressed adipogenesis and mitochondrial dysfunction specific to PLWH, reduced telomere length specific to the elderly). Given the partial signs of ageing-related alterations in adipose tissue from PLWH, existing nutritional and treatment-based procedures to avoid accelerated ageing, although limited, should be considered for PLWH affected by lipodystrophy. Further longitudinal studies in ageing PLWH are warranted to ascertain whether their signs of aged adipose tissue (and the related pathogenic consequences) worsen and accelerate with ageing, even in the absence of overt lipodystrophy.

## Supplementary Material

Supplemental Material

## Data Availability

The data that support the findings of this study are openly available in Adipoplast website at https://adipoplast.org/datebase/
