# Supplementary material for: Adipose tissue aging partially accounts for fat alterations in HIV lipodystrophy
Source: Adipocyte. 2022 Mar 17;11(1):143–52. doi: 10.1080/21623945.2022.2042962 (PMC8942446; doi:10.1080/21623945.2022.2042962)
Supplement: Supplemental Material [file KADI_A_2042962_SM3618.zip › Supplementary tables.docx]

**Supplementary Table 1**. Plasma viral load, CD count and antiretroviral drug exposure of studied HIV-1 infected patients

|  | ART treated HIV-1 infected patients |
| --- | --- |
| Plasma viral load | 141.11 ± 111.71 |
| CD4 count (cells/mm^3^) | 904 ± 96.91 |
| CD4% | 33.35 ± 2.36 |
| CD8 count (cells/mm^3^) | 1014 ± 112 |
| CD8% | 37.41 ± 2.71 |
| NRTI (months) | 394.66 ± 9.53 |
| AZT (zidovudine) | 34.62 ±10.49 |
| d4T (stavudine) | 65.92 ± 10.74 |
| 3TC (lamivudine) | 60.07 ± 10.61 |
| ddI (didanosine) | 69.80 ± 15.07 |
| ddC (zalcitabine) | 22 ± 0.0 |
| ABC (abacavir) | 40 ± 13.92 |
| TDF (tenofovir) | 61.11 ± 8.11 |
| FTC (emtricitabine) | 41.14 ± 6.36 |
| NNRTI (months) | 119.67 ± 10.64 |
| Efavirenz | 68.77 ± 14.22 |
| Nevirapine | 44.51 ± 15.03 |
| Etravirine | 6.42 ± 2.69 |
| PI (months) | 249.24 ± 9.92 |
| Indinavir | 14.78 ± 6.26 |
| Saquinavir | 23.57 ± 9.77 |
| Ritonavir | 62.03 ± 12.75 |
| Nelfinavir | 11.52 ± 5.66 |
| Lopinavir | 62.01 ± 17.66 |
| Atazanavir | 7.62 ± 4.95 |
| Fosamprenavir | 20.33 ± 20.33 |
| Darunavir | 22.58 ± 5.53 |
| Raltegravir | 24.88 ± 6.35 |

Cumulative months of drug exposure are expressed as mean ± SEM. NRT, nucleoside analog reverse transcriptase inhibitor; NNRTI, non-nucleoside analog reverse transcriptase inhibitor; PI, protease inhibitor.

**Supplementary Table 2.** TaqMan RT-PCR gene expression assays

|  | Reference code |
| --- | --- |
| TNF (TNFα) | Hs00174128_m1 |
| CXCL8 (Interleukin-8) | Hs00174103_m1 |
| CCL2 (MCP-1) | Hs00234140_m1 |
| CEBPA | Hs00269972_s1 |
| ADIPOQ | Hs00605917_m1 |
| LEP | Hs00174877_m1 |
| PPARG | Hs01115513_m1 |
| MT-CO2 | Hs02596865_g1 |
| COX4l1 | Hs00971639_m1 |
| SLC2A1 (GLUT1) | Hs00892681_m1 |
| SLC2A4 (GLUT4) | Hs00168966_m1 |
| CDKN1A (P21^WAF-1^) | Hs00355782_m1 |
| CDKN2A (P16^INK4a^) | Hs00923894_m1 |
| TP53 (p53) | Hs01034249_m1 |
| MDM2 | Hs00540450_s1 |
| ULK1 | Hs00177504_m1 |
| ATG4A | Hs00364702_m1 |
| ATG4D | Hs00262792_m1 |
| GABARAP | Hs00925899_g1 |
| BNIP3 | Hs00969291_m1 |
| PARK2 | Hs01038318_m1 |

**Supplementary Table 3.** Primer sequences used to quantify telomere repeats

|  | Primer sequence |
| --- | --- |
| Telomere forward | 5’-CGG TTT GTT TGG GTT TGG GTT TGG GTT TGG GTT TGG GTT-3’ |
| Telomere reverse | 5’-GGC TTG CCT TAC CCT TAC CCT TAC CCT TAC CCT TAC CCT-3’ |
| 36B4 (control) forward | 5’-GCA AGT GGG AAG GTG TAA TCC-3’ |
| 36B4 (control) forward | 5’-ATT CTA TCA TCA ACG GGT ACA A-3’ |
